# Regulatory B Cells and Tolerance in Transplantation: From Animal Models to Human

**DOI:** 10.3389/fimmu.2013.00497

**Published:** 2013-12-31

**Authors:** Mélanie Chesneau, Laure Michel, Nicolas Degauque, Sophie Brouard

**Affiliations:** ^1^Institut National de la Santé et de la Recherche Médicale U1064, Institut de Transplantation Urologie Néphrologie, Nantes, France; ^2^Université de Nantes, Nantes, France; ^3^Centre Hospitalier Universitaire, Nantes, France

**Keywords:** transplantation, regulatory B cells, animal, human, treatment, tolerance

## Abstract

Until recently, the role of B cells in transplantation was thought to be restricted to producing antibodies that have been clearly shown to be deleterious in the long-term, but, in fact, B cells are also able to produce cytokine and to present antigen. Their role as regulatory cells in various pathological situations has also been highlighted, and their role in transplantation is beginning to emerge in animal, and also in human, models. This review summarizes the different studies in animals and humans that suggest a B-cell regulatory role in the transplant tolerance mechanisms.

## Introduction

Reducing immunosuppressive drug doses is one of the major goals in transplantation. In liver transplantation, up to 20% of recipients could eventually be weaned off immunosuppression (1, 2). Although the kidney is less disposed to successful immunosuppressive drug withdrawal, more than a hundred cases of operationally tolerant (OT) renal transplant patients have been reported (2–5). These are patients who display continued good graft function in the absence of immunosuppressive drugs (6). Understanding the mechanisms involved in these patients would provide invaluable information about the pathways to human transplantation tolerance.

Over recent decades, tremendous progress has been made in the understanding of the biology of regulatory cells and their roles in autoimmunity, infection, cancer, and transplantation. The best-known are the CD4^+^Foxp3^+^ T cells, and their role in transplantation, at least in animal models, has been clearly established (7). Until recently, the role of B cells in transplantation was thought to be restricted to producing antibodies that have been clearly shown to be deleterious in the long-term (8, 9), but B cells are also able to produce cytokine and to present antigen (10, 11). Their role as regulatory cells has also been highlighted in varying pathological situations and their role in transplantation has begun to emerge. The present review will focus on the role of these cells in transplantation, in animal models and in clinic.

## Regulatory B Cells: Phenotype and Mechanisms of Action

While a suppressive role for B cells was already suspected in the mid-70s in a model of delayed hypersensitivity in guinea pigs (12, 13), it is in 1996 that Wolf et al. (14) really pointed toward the existence of “regulatory” B cells. Unlike wild type mice, B-cell deficient mice (μMT) were unable to recover from Experimental Autoimmune Encephalomyelitis (EAE) and this was later attributed to the absence of IL-10-producing B cells. Since this first report, the major role of these cells has been reported in numerous autoimmune disease models. As was the case for regulatory T cells (Treg) in the 1980s, there are, as yet, no validated phenotypic markers of regulatory B cells, and it remains very likely that, as for Treg cells, different regulatory B-cell subsets exist. Two main B-cell populations have been reported in mice. The precursor B cells of the marginal zone (T2-MZP B cells) were described by Evans et al. (15) in a collagen-induced arthritis (CIA) mouse model. These cells produce IL-10, have a CD19^+^CD21^high^CD23^+^CD24^high^CD93^+^ phenotype, and their adoptive transfer from naïve mice to immunized mice suppresses CIA development in an IL-10 dependent manner. Tedder and colleagues identified another subset of regulatory B cells in mice based on IL-10 expression, called B10 cells. This rare B-cell subset (1–2%) is found predominantly in the spleen CD1d^high^CD5^+^ B-cell subset of naïve wild type mice and is defined by its unique capacity to produce IL-10 in response to specific activation signals (16). In a contact hypersensitivity (CHS) model of inflammation, Tedder’s group has shown that the B10 cells suppress T-cell dependent inflammation during CHS *in vivo* in an antigen-dependent manner (17). Even if there are significant phenotypic differences between these two B-cell subsets, it cannot yet be excluded that they share a common progenitor. More recently, studies have demonstrated that a combination of IL-21 and B-cell antigen receptor (BCR) stimulation enables B cells to produce and secrete Granzyme B, without the secretion of perforin. This Granzyme B secretion by B cells may also play a major role in the regulation of autoimmune responses (18). So different subsets of regulatory B cells seem to exist with, most likely, different mechanisms of action.

Concerning the activation of Bregs, several studies demonstrate the major role of CD40 pathway stimulation for Breg IL-10 secretion (19, 20) and also the involvement of Toll Like Receptors (TLRs) (16, 17, 21). Interestingly, Yanaba et al. showed as recently as last year that B10-cell maturation into functional IL-10-secreting effector cells requires IL-21 and CD40-dependent cognate interactions with T cells (22). Some studies have also shown that the regulatory function of B cells was antigen specific in an EAE and in a CHS model (16, 23), and also that these Bregs can differentiate into plasmocytes and plasmablasts secreting poly-reactive or antigen-specific antibodies (24). Recently Montandon et al. also described a new population of B cells with regulatory properties in an animal model of type-1 diabetes. These are a hematopoietic progenitor population: innate pro-B cells which protect non-obese diabetic mice against type-1 diabetes. Pro-B cells activated by TLR-9 suppress pathogenic effectors cells by reducing their IL-21 production and by inducing apoptosis via Fas Ligand (25).

**Figure 1:**
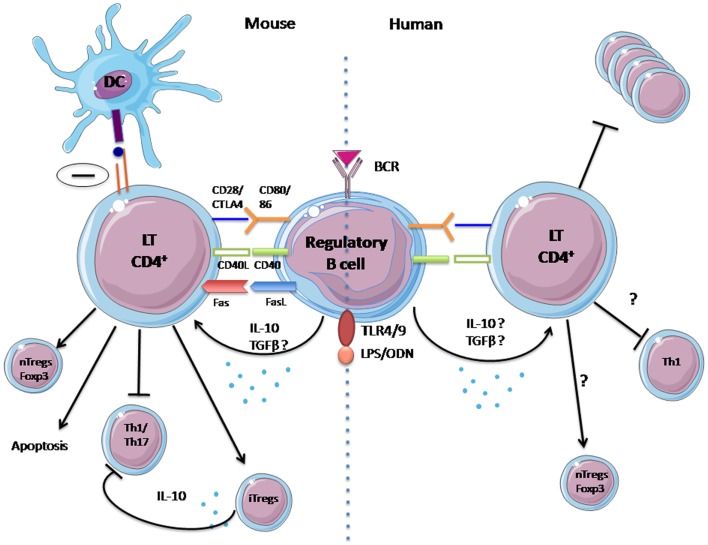
**Mechanisms of suppression of regulatory B cells identified in human and animal**. In mice, regulatory B-cell suppression is fulfilled by IL-10 secretion, activation of the CD40 pathway, and probably via contact with T lymphocytes. It has numerous effects: (1) inhibition of Th1 and Th17 differentiation, (2) inhibition of antigen presentation by DCs, and (3) induction of natural regulatory T cells. For humans, the mechanisms for the actions of regulatory B cells remain unclear and have yet to be confirm: (1) Probable inhibition of proliferation of CD4^+^ T cells, (2) Possible inhibition of Th1 differentiation, and (3) possible increase of natural regulatory T cells.

Similarly to Tregs, Bregs exert their suppressive properties in different ways: Th1 and Th17 differentiation inhibition (15, 19, 20, 23, 26–28) regulatory T-cell induction (28–30); and also through a direct inhibitory effect on antigen presentation by DC (23). These suppressive mechanisms are summarized in Figure 1.

In humans, these regulatory B cells have recently been identified and described. However, their study is still in its infancy and their phenotype needs to be better described. Blair et al. (26) demonstrated that human transitional CD19^+^CD38^hi^CD24^hi^ B cells possess regulatory capacities (31). This has also been confirmed in healthy volunteers by Lemoine et al. (32). After CD40 stimulation, these cells suppress the differentiation of T helper 1 cells, partially via the provision of IL-10. Their suppressive capacity is reversed by a blockade with CD80 and CD86 monoclonal antibodies, suggesting a contact-dependent suppressive action. In 2010, the group of Tedder characterized IL-10 competent B cells in humans. They describe a B10 subset defined by its capacity to secrete IL-10 after 5 h of *ex vivo* stimulation, whereas progenitor B10 (B10pro) cells require 48 h of *in vitro* stimulation before they acquire the ability to express IL-10 (33). Both subsets are predominantly found within the memory CD24^hi^CD27^+^ B-cell subpopulation and are able to negatively regulate monocyte cytokine production through IL-10 dependent pathways during *in vitro* functional assays. In addition, a recent study demonstrated that human B cells can regulate DC maturation and function (34).

AS can be seen from the above, currently the majority of studies looking at Bregs in human autoimmune diseases. However, studies in the area of transplantation have produced a number of arguments pointing to a major implication of B cells in tolerance. The following will focus on the role of Bregs first in animal tolerance models, and then in human.

## Part I: Regulatory B Cells in Animal Model of Transplantation

The following provides a review of experimental models demonstrating the implication of B cells as major actors in inducing tolerance (Table 1).

**Table 1:** **Summary table of studies demonstrating the implication of B cells as major actors in tolerance induction in different kinds of experimental animal models**.

Reference	Animal model	Modality of tolerance	Implication of B cells
Parker et al. (35)	Mouse pancreatic islet allografts	Treatment with allogenic small lymphocyte or T-depleted small lymphocytes plus blocking antibody to CD40L	Increase survival of recipients treated with T-depleted small lymphocytes plus CD40L
Niimi et al. (36)	Mouse model of cardiac allograft	Resting B cells plus blocking antibody to CD40L	Tolerance induced by B cells involves the CD40 pathway
Yan et al. (37)	Rat model of kidney allograft	I.V. injection of donor B cells at time of transplantation	B cells induce more efficiently long-term acceptance of graft than T cells
Deng et al. (38)	Mouse model of cardiac allograft	Anti-CD45-RB therapy	Anti-CD45-RB is not efficient in transgenic mouse without B cells
Huang et al. (39)	Mouse model of cardiac allograft	Treated with anti-CD45-RB, anti-ICAM, anti-LFA or combination of these agents	Expression of ICAM-1 by B cells and interaction with LFA-1 form a central aspect of transplantation tolerance induced by CD45-RB therapy
Zhao et al. (40)	Mouse model of cardiac allograft	Anti-CD45-RB therapy	IL-10 expressed by B cells inhibits B-cell-mediated tolerance induction in cardiac allograft model
Ding et al. (41)	Mouse model of islet allograft	Anti-TIM-1 therapy	TIM-1 B cells are regulatory and transfer donor-specific long-term graft survival
Le Texier et al. (42)	Rat model of cardiac allograft	Short-term immunosuppression	Accumulation of B cells in PBMC of tolerant recipients and a phenotype of inhibited B cells partially blocked at their IgM to IgG switch and over expressing the inhibitory receptor Fcgr2b
Lee et al. (43)	Mouse model of islet allograft	Anti-CD45-RB and anti-TIM-1 therapy	Combined anti-CD45-RB and anti-TIM-1 treatment induced allograft survival that is B-cell dependent, dependent on B-cell production of IL-10, and is associated with up-regulation of TIM-1 on B cells

The first evidence for a potential role for B cells in allograft tolerance was reported by Parker et al. (35). In a pancreatic islet allograft BALB/c mouse model, survival of C57Bl/6 recipient mice was increased by injection of a large quantity of B cells, in addition to a CD40 ligand (CD40L) blocking antibodies to prevent T-cell/B-cell interaction, 8 days before islet transplantation [from (BALB/C × C57BL/6)F1). Allogenic donor B cells thus permit islet allograft survival when administrated in combination with anti-CD40L (35).

Niimi et al. (36) confirmed the role of B cells in the tolerance induction after blockade of CD40L-CD40 interaction in a cardiac allograft mouse model. They induced tolerance to cardiac allograft in C3H mice by treating the recipients with a donor-specific subset of B cells (resting B cells that are incompetent or non-professional APCs) from C57BL/6 mice and blocking CD40L 14 days prior to graft. Furthermore, induction of tolerance by resting B cells was abrogated in CD40L Knockout mice, confirming that the CD40 pathway plays a critical role in allograft rejection *in vivo* (36).

Yan et al. (37) demonstrated in DA recipients of a kidney allograft from PVG rat, that donor B-cell administration at the time of transplantation induces long-term acceptance more efficiently than donor T cells. However, the mechanism by which B cells induce long-term allograft survival was not elucidated (37). Other studies have also pointed to a role for B cells in inducing tolerance by immunosuppression targeting CD45 (38–40). CD45 is part of family of transmembrane protein tyrosine phosphatases involved in lymphocyte development and activation (51) and serves as a rheostat determining the threshold of antigen stimulation. Deng et al. have shown that short-term administration of anti-CD45-RB antibodies on days 0, 3, 5, and 7 following transplantation efficiently prevents cardiac allograft rejection in both the allogeneic C3H to B6 and the BALB-derived transgenic HA104 [Hemagglutinin (HA) expressing] to TS1 (HA specific TCR) combination (38). In this model, tolerance induced by anti-CD45-RB was lost in B6μMT^−/−^ mice, a model of transgenic mouse lacking B cells and antibodies. Tolerance was restored after B-cell transfer in B6μMT^−/−^ mouse, showing that tolerogenic efficacy of anti-CD45-RB therapy requires host B cells. Long-term survival was not obtained when cardiac allografts were transplanted to B-cell deficient mice reconstituted with splenocytes from knockout mice with a deficiency for the co-stimulatory molecule CD40 or the CD80/CD86 combination. These data show that tolerance induced by anti-CD45-RB therapy requires host B cells and that tolerance is mediated through the interaction of co-stimulatory molecules on B cells and T cells (38). In the same area of research, Huang et al. have shown that, in a model of cardiac allograft from C3H donors into B6 recipient mice, anti-CD45-RB Ab therapy on days 0, 1, 3, 5, and 7 after transplantation induce tolerance. Splenic B-lymphocytes demonstrate phenotypic alterations including up-regulation of CD54 (intracellular ICAM-1 adhesion molecules) (52). Blockade of ICAM-1/LFA-1 interaction prevents the tolerance induction by anti-CD45-RB and mice deficient in either ICAM-1 or LFA-1 reject their graft even when they are treated with anti-CD45-RB (39). In their study, Zhao et al. investigated the role of IL-10 in an anti-CD45-RB model of mouse cardiac allograft. Surprisingly, in this model, neutralization of IL-10 by treatment with five doses of anti-IL-10 antibody every other day post transplantation improves tolerance induction. The role of B-cell IL-10 production was assessed by transferring IL-10 deficient splenocytes into B-cell deficient recipients in which tolerance could be then induced in a similar manner as in the IL-10 non-deficient splenocytes, confirming that Il-10 expression by B-lymphocytes inhibits B-cell-mediated tolerance induction. Neutralization of IL-10 enhances tolerance induction and improves the long-term outcomes of cardiac allograft (40).

Due to the lack of regulatory B cells markers, certain research focuses on the development of phenotypic markers. T-cell immunoglobulin and mucin domain (TIM) family proteins are potent co-stimulatory molecules in T-cell activation (53). Anti-mouse TIM-1 mAb RMT1-10 given i.p. on day of transplantation at 0.5 mg and on days 2, 4, 6, 8, and 10 after transplantation at 0.25 mg prolongs graft survival in one mice model of cardiac allograft (B6 to BALB/C) (54). Ding et al. (41) showed that anti-TIM-1 (RMT1-10) administered i.p. on days 1, 0, and 5 relative to day of islet (B6) transplantation to Balb/c prolong islet allograft survival. Depletion of B cell (anti-CD20) in recipients prior to transplantation shortened allograft survival compared with B-cell-intact mice, demonstrating that B-lymphocytes are required for prolonged anti-TIM-1-reliant allograft survival. In this model, both islet transplantation and the treatment of the recipient with anti-TIM-1 increased IL-10 and IL-4 expression on B cells. The expression of these two cytokines in TIM-1^+^ vs. TIM-1^−^ B cells show that TIM-1^+^ B cells expressed more IL-4 and IL-10 than other B cells. In this study, TIM-1^+^ and TIM-1^−^ B cells from splenic B cells of Balb/c allograft recipients treated with anti-TIM-1 and sacrificed on day 14 were sorted. Transfer of TIM-1^+^ B cells into untreated JHD recipients of B6 islet prolongs allograft survival whereas TIM-1^−^ B cells have no effect, demonstrating that TIM-1^+^ B cells have a regulatory role and are able to transfer donor-specific long-term graft survival properties (41).

Lee et al. studied the effect of anti-CD45-RB treatment in combination with anti-TIM-1 antibodies. This tolerance induction in a mouse islet allograft model of C57BL/6 diabetic recipients and islets from BALB/C demonstrates that this model is dependent on the production of IL-10 by B cells. Transfer of WT B cells prolong graft survival whereas IL-10 deficient B cells do not prolong allograft survival, suggesting that graft survival is dependent on IL-10 production by B cells in this model. Depletion of Treg by anti-CD25 PC61 before transplantation leads to a rejection of allograft suggesting that tolerance induction is also dependent on TIM-1^+^ regulatory B-cell/Treg interaction (43).

In a LEW1W/LEW1A heart allograft rat model, administration of LF15-0195, an analog of deoxyspergualin, for 20 days starting at transplantation induces long-term cardiac allograft tolerance (42). The tolerated allograft contains B cells organized in germinal centers with strongly inhibited IgM to IgG switch. The authors report on an accumulation of B cells in the blood of tolerant recipients following cessation of immunosuppressive treatment (at days 30 and 100 after transplantation). Blood B cells from tolerant recipients express a lower ratio of IgG/IgM transcripts and display an over-expression of BANK-1 and FcgR2b compared with B cells from recipients that develop chronic rejection. BANK-1 is an adapter protein involved in B-cell receptor-mediated signaling that negatively regulates CD40-mediated AKT activation (55) and the inhibitory receptor Fcgr2b is a member of the immunoreceptor tyrosine-based activating/inhibitory motif (ITAM/ITIM) family. Colligation of FcgammaRIIb with the BCR results in the abrogation of B-cell activation. Fcgr2b may play a role in the maintenance of peripheral tolerance and its polymorphism is, like that of BANK-1, significantly associated with systemic lupus erythematosus (SLE) (56, 57). This result may reflect an accumulation of inhibitory or inhibited B cells blocked in their switch recombination process. These data suggest that the B cells may not have proceeded to somatic hypermutation and therefore may produce Donor-Specific Antibody (DSA) of low affinity.

Human Alpha-1-Antitrypsin (hAAT) is a clinically available anti-inflammatory circulating glycoprotein known to protect islets from allorejection through the expansion of Tregs and alteration of dendritic cell responses (58, 59). A recent study from Mizrahi et al. demonstrates that B cells could participate toward tolerance in allogeneic transplantation via hAAT. First, they demonstrate that, *in vitro*, hAAT reduces B-cell activation in LPS-stimulated culture by lower expression of activation markers CD40 and CD19 and release of IL-10, and also affects T-cell-dependent B-cell activation by reducing expression of co-stimulatory molecules in B cells (CD80 and CD86) upon stimulation with CD40L. They demonstrated that the presence of IL-10-producing B cells is elevated by hAAT. They also show that B-cell knockout hATT transgenic chimeric mice fail to exhibit the increase in Treg observed in hAAT transgenic mice, suggesting a role for B cells in the induction of Treg by hAAT. This study brings to light the potential regulatory function of B cells in the protection of islet allorejection induced by hAAT.

## Part II: Regulatory B Cells in Human Tolerance

The following provides a review of studies demonstrating the implication of B cells in kidney transplant operational tolerance (Table 2). Despite tolerance now being commonly obtained in animal models, it remains a challenge in clinic. In humans, operational tolerance has been defined as a stable graft function without clinical features of chronic rejection in the absence of any immunosuppressive drugs for more than 1 year (6). A creatinemia and proteinuria below 150 μmol/l and 1 g/24 h respectively have been defined as acceptable thresholds. This definition has been accepted by the two main European and American consortia^1^^,^^2^. Even if the experiments are performed in the peripheral B cells, there is a certain amount of research indicating a possible involvement of these cells in the mechanisms of tolerance in OT patients. A study, published in 2006, reported that chronic rejection recipients display a significantly lower absolute number of B cells compared to tolerant recipients (44). One year later, we described a specific, sensitive peripheral transcriptional signature in OT patients. Inside the footprint of the 49 genes identified, several genes implicated B cells, such as CD79a, b, CD19, and CD20 (45). Following this study, we reported on an increase in both the absolute number and frequency of total B cells in the blood from 12 tolerant kidney-transplant recipients, due in particular to an expansion of activated and memory B cells. These cells had an inhibitory phenotype, defined by the increased expression of BANK-1 (which negatively modulates CD40-mediated AKT activation), CD1d, and CD5, as well as a decreased proportion of CD32a/CD32b (46). The same year, Newell et al., sponsored by the Immune Tolerance Network (ITN), studied 25 tolerant kidney-transplant recipients. They sought to identify immunity parameters to discriminate tolerant recipients from subjects with stable allograft under immunosuppression, as well as healthy controls (4). This study included the identification by microarray and real-time PCR of 30 genes, over two thirds of which are B-cell specific, that distinguish between tolerant and non-tolerant individuals. Most notably, the tolerant cohort differentially expressed three B-cell genes (IGKV4-1, IGLL1, and IGKV1D-13) that proved to be highly predictive of tolerance in a new set of patients. Moreover, the analysis of the phenotype of peripheral blood B cells revealed an increase in naïve and transitional B cells in the tolerant group. Finally, the stimulated transitional B cells from tolerant recipients produced more IL-10 *in vitro* compared to the non-tolerant group, even if this secretion remained very low. As Il-10 is one of the main features of regulatory B cells, these data suggest that OT patients could present a larger subset of regulatory B cells than the non-tolerant patients (4). Recently Newell et al. showed that IGKV1D-13 appears as the most stable and discriminating gene and propose this single gene as a signature of operational tolerance (Personal communication, ATC, Boston, 2012).

**Table 2:** **Summary table of studies demonstrating the implication of B cells in kidney-transplant operational tolerance**.

Reference	Groups studied	Implication of B cells
Louis et al. (44)	TOL/STA/CR	TOL display more circulating B cells compared to STA and CR
Brouard et al. (45)	TOL/STA/CR/HV	Transcriptional signature in OT patients. Footprint of 49 genes, several genes implicated B cells, CD79a,b, CD19, CD20
Pallier et al. (46)	TOL/STA/CR/HV	Higher absolute number and frequency of total B cells in blood. Increased expression of BANK-1, CDld, CD5, FCyRllb in TOL vs. STA
Newell et al. (4)	TOL/STA/HV	B-cell signature in TOL patients, increase of naives B cells and increase of IL-10 expression in TOL vs. STA
Sagoo et al. (5)	TOL/STA/CR/HV	Increase number of B cells and TGF-b producing cells in TOL vs. STA and CR
Danger et al. (47)	TOL/STA	Over-expression of miR142-3p in B cells and increase of TGF-Bl expression in B cells from TOL vs. STA
Silva et al. (48)	TOL/STA/CR/HV	Transitional B cells from TOL preserved ability to activate the CD40/STAT3 signaling pathways in transitional B cells in contrast with CR
Haynes et al. (49)	TOL/STA/CR	Increase of circulating naive B cells in TOL vs. STA and CR. Higher POT score (“probability of being tolerant”: score including B-cell parameters and direct pathway T-cell parameters) in TOL vs. STA and CR
Chesneau et al. (50)	TOL/STA/HV	Less plasma cells in TOL vs. STA. *In vitro* their is a default in B-cell differentiation and an increase B-cell sensitivity to apoptosis in late step of differentiation of B cells from TOL vs. STA. Increase of IL-10 expression by activated B cells in TOL vs. STA

Another multicenter study, including 11 tolerant kidney-transplant recipients conducted by the RISET consortium reported similar results: they also found over-expression of B-cell related genes in OT patients compared to the other groups of patients (stable patients, chronic rejection, and healthy controls) by microarray analysis, and a displayed increase in B cells and NK cells (5). Interestingly, they also reported a relative increase in TGF-β producing cells in OT patients.

Taken together, these findings show that OT patients have a particular blood B-cell phenotype and suggest that B cells may participate in or contribute to the maintenance of long-term graft function in these patients. These three studies were partly validated by a recent study describing a significant increase in circulating naïve B-cell numbers in OT patients compared to stable treated patients or chronically rejecting patients, with a higher POT score (“probability of being tolerant”: score including B-cell parameters and direct pathway T-cell parameters (5), in the OT group (49). However, the donor-specific indirect pathway analysis in this study revealed a B-cell independent, IL-10 independent but TGF-b dependent signature in the OT group (49). So, the precise mechanisms by which B cells induce tolerance remain elusive and are not as clear as in animal models. Interestingly, among the specific blood signature of the 49 genes associated with tolerance, 27% of the genes modulated in the blood of OT patients could be regulated by the TGF-b (45). We ourselves recently showed that miRNA 142-3-p is up-regulated in B cells of OT patients with a stable expression over time. This study suggests that a negative feedback loop involving TGF-β signaling and miR-142-3p expression in B cells may contribute to the maintenance of tolerance (47).

A recent study reported a preserved BCR repertoire in OT patients, similar to that in healthy individuals (48). In addition, tolerant patients also displayed a conserved capacity to activate the CD40/STAT3 signaling pathways in transitional B cells, in contrast to patients with chronic rejection. The authors conclude that the B-cell regulatory compartment is preserved in OT patients (48). However, these results need to be confirmed, the numbers of OT patients being low (*n* = 5).

A recent study by Nouël et al. studied B cells in chronic rejection. They demonstrated that B cells from chronic rejection patients are unable to efficiently inhibit autologous T-cell proliferation, as B cells from stable patients or healthy volunteers can. Indeed, B-cell inducing tolerance could be explained by a preserved B-cell compartment (60).

More recently, B cells in operational tolerance in kidney transplantation was also analyzed in the study of Chesneau et al. In this study, we show that B cells from tolerant patients display a lack of plasma cells compared to stable patients that may be due to a default in *in vitro* B-cell differentiation and an increase of B cells sensibility to apoptosis in late step of differentiation of B cells from tolerant patients. Furthermore in this study we show that activated B cells from tolerant patients secrete more IL-10 compared to healthy volunteers and stable patients. This study reinforce the potential regulatory properties of B cells in tolerant patients with an over-expression by B cells of IL-10 after *in vitro* stimulation in tolerant compared to healthy volunteers and stable patients and a default in plasma cell/naives cells balance in tolerant compared to stable patients (50).

Finally, in a model of tolerance induction based on a combined kidney and bone marrow transplantation in humans, Porcheray et al. showed that in three-quarters of tolerant patients, *de novo* antibodies specific to donor antigens and/or C4d deposition in the graft developed. This antibody response coincided with B-cell reconstitution and a high frequency of peripheral transitional B cells (61). The involvement of B cells in the mechanisms of tolerance is not limited to OT patients but also concerns also patients with therapeutic induced tolerance.

## Conclusion

Interestingly, in contrast to kidney tolerant recipients, in liver transplantation around 20% of patients can be successfully weaned off immunosuppression (62, 63). Contrary to kidney-transplant patients, these liver tolerant patients do not present an increase in the absolute numbers of peripheral blood B-lymphocytes, no modification of the different subsets of B cells and no B-cell transcriptional pattern but a NK cell signature (64). These data suggest that the mechanisms involved in the induction and maintenance of this tolerance process remain as yet undetermined and probably differs depending on the kind of organ transplanted. In kidney transplantation, whether these regulatory B cells are a driving force for tolerance induction, or whether they simply help to stabilize tolerance in the absence of immunosuppression, has not been established. Since the first cases of OT were described, the problem of inadequate comparators has remained unsolved. This paradox is due to the clinical situation of patients who display stable graft function but no longer receive immunosuppression. Healthy volunteers share with these patients the absence of immunosuppression but have not received a transplant, whereas stable patients share graft function stability but are under immunosuppression. This absence of adequate controls remains a difficulty in the comparison immunological parameters.

Nonetheless, all these data lead us to increasing exploration of various therapeutic approaches to inducing tolerance by promoting the development of B cells with regulatory functions. In fact, similarly to Treg, studies suggest that regulatory B cells present varying abilities to suppress immune responses depending on the environment, on the trigger, and probably by using different mechanisms (65). These characteristics will have to be considered and studied, before developing new therapeutic strategies.

## Conflict of Interest Statement

The authors declare that the research was conducted in the absence of any commercial or financial relationships that could be construed as a potential conflict of interest.

## Footnotes

^1^www.risetfp6.org/

^2^http://www.immunetolerance.org/In
